# Different bacterial communities in heat and gamma irradiation treated replant disease soils revealed by 16S rRNA gene analysis – contribution to improved aboveground apple plant growth?

**DOI:** 10.3389/fmicb.2015.01224

**Published:** 2015-11-06

**Authors:** Bunlong Yim, Traud Winkelmann, Guo-Chun Ding, Kornelia Smalla

**Affiliations:** ^1^Section of Woody Plant and Propagation Physiology, Institute of Horticultural Production Systems, Leibniz Universität HannoverHannover, Germany; ^2^College of Resources and Environmental Sciences, China Agricultural UniversityBeijing, China; ^3^Beijing Key Laboratory of Biodiversity and Organic farming, China Agricultural UniversityBeijing, China; ^4^Institute for Epidemiology and Pathogen Diagnostics, Julius Kühn-Institut – Federal Research Centre for Cultivated PlantsBraunschweig, Germany

**Keywords:** biotest, apple replant disease, DGGE, qPCR, pyrosequencing, bacterial community composition, bacterial diversity

## Abstract

Replant disease (RD) severely affects apple production in propagation tree nurseries and in fruit orchards worldwide. This study aimed to investigate the effects of soil disinfection treatments on plant growth and health in a biotest in two different RD soil types under greenhouse conditions and to link the plant growth status with the bacterial community composition at the time of plant sampling. In the biotest performed we observed that the aboveground growth of apple rootstock M26 plants after 8 weeks was improved in the two RD soils either treated at 50°C or with gamma irradiation compared to the untreated RD soils. Total community DNA was extracted from soil loosely adhering to the roots and quantitative real-time PCR revealed no pronounced differences in 16S rRNA gene copy numbers. 16S rRNA gene-based bacterial community analysis by denaturing gradient gel electrophoresis (DGGE) and 454-pyrosequencing revealed significant differences in the bacterial community composition even after 8 weeks of plant growth. In both soils, the treatments affected different phyla but only the relative abundance of *Acidobacteria* was reduced by both treatments. The genera *Streptomyces, Bacillus, Paenibacillus*, and *Sphingomonas* had a higher relative abundance in both heat treated soils, whereas the relative abundance of *Mucilaginibacter, Devosia*, and *Rhodanobacter* was increased in the gamma-irradiated soils and only the genus *Phenylobacterium* was increased in both treatments. The increased abundance of genera with potentially beneficial bacteria, i.e., potential degraders of phenolic compounds might have contributed to the improved plant growth in both treatments.

## Introduction

Intensive production of perennial and annual crops in the same area might lead to replant problems which become evident by low yields and growth reduction (Hoestra, 1994). The replant problems are potentially caused by both biotic and abiotic factors (Hoestra, 1994; Politycka and Adamska, 2003). The decline in plant growth evoked by biotic factors is called “replant disease” (RD; Utkhede, 2006). The RD was already reported by Klaus (1939), and the disease incidence is typically resident and persistent. It is most likely that biotic factors including soilborne pathogens play a major role since significantly improved growth after heat or chemical soil treatment was reported compared to the growth in untreated soil (Mai and Abawi, 1978; Hoestra, 1994; Parchomchuk et al., 1994; Utkhede, 2006; Yim et al., 2013). The important role of soilborne organisms in apple RD was recently discussed in a review by Mazzola and Manici (2012). As reported in various studies, possible causes of apple RD differed widely between regions and included actinomycetes (Westcott et al., 1986; Otto et al., 1994), *Pythium* sp. (Hoestra, 1994; Emmett et al., 2014), *Cylindrocarpon* sp., *Phytophthora* sp. and *Rhizoctonia solani* (Mazzola, 1998; Tewoldemedhin et al., 2011; Kelderer et al., 2012) as well as nematodes, e.g., *Pratylenchus penetrans* (Mai et al., 1994).

Bacteria and fungi associated with apple RD were traditionally identified after isolation from the respective soils. However, the soil microbial diversity is highly complex and can be only partially evaluated by traditional cultivation techniques as a large proportion does not form colonies on solid media after plating. It was estimated that less than 14% of the bacterial cells per gram of soil can be cultured (Janssen et al., 2002; Janssen, 2006). The analysis of soil total community (TC-) DNA or RNA helped to overcome this limitation. Gomes et al. (2005) reported no pronounced differences between DNA and cDNA based fingerprints when working with soils. In many studies, fingerprinting and amplicon sequencing methods have been applied to study soil bacterial communities based on 16S rRNA gene fragments amplified from soil TC-DNA (reviewed by Berg and Smalla, 2009).

In the study by Yim et al. (2013), a biotest was developed to determine the degree of apple RD in a given soil. It involved soils untreated and treated at 50°C and at 100°C. It is assumed that the treatment at 50°C primarily affects nematodes, bacteria and fungi sensitive to this temperature, while the treatment at 100°C strongly reduces the total soil microbiota (Pullman et al., 1981; Cabos et al., 2012). A comparison of the growth of *in vitro* propagated apple rootstock M26 in these three soil variants under greenhouse conditions for 10 weeks clearly indicated the level of the RD. The DGGE analysis of 16S rRNA gene fragments amplified from soil TC-DNA revealed a distinct bacterial community composition of the soils depending on the treatments at the end of the biotest (Yim et al., 2013); the DGGE fingerprints did not provide more detailed information on the taxonomy of bacterial responders.

In the present study, a modified biotest was employed by using a gamma irradiation treatment instead of a heat treatment at 100°C since gamma irradiation has less influence on soil physical and chemical properties (Trevors, 1996). This study aimed to investigate the effects of soil disinfection treatments on plant growth and health in a biotest in two different RD soil types and to link the plant growth status with the bacterial community composition and diversity at the time of plant sampling. We hypothesized that differences in apple rootstock growth and symptoms observed resulted from changes in the microbial community composition and diversity in RD soils caused by their heat or gamma irradiation treatment. At the end of the biotest, the bacterial community composition of soil loosely adhering to the roots was analyzed by DGGE and pyrosequencing of 16S rRNA gene fragments amplified from TC-DNA. Statistical analysis of the 454 pyrosequencing data allowed us to identify responders to the treatments. In addition soil samples taken before the biotest were included in the DGGE analyses.

## Materials and Methods

### Soil Characteristics

Two RD soils were obtained from two private nurseries, Kle (53°41′ 58.51″ N, 9° 41′ 34.12″ E) and Alv (53° 42′ 18.81″ N, 9° 48′ 16.74″ E) in the Pinneberg area in Germany. Both soil types had different cropping and management histories. The Kle site soil had been mainly cultivated with rose rootstock plants from 1980 until 2011, and crop rotation with *Tagetes* started in 2002. In the Alv soil, apple rootstock plants had been planted for several years until 2009. Then, *Prunus domestica* and *Cydonia oblonga* were grown in 2010 and 2011, respectively. In May 2012, the apple rootstock ‘M4’ was planted in both soils. Supplementary Table S1 shows the characteristics of both soil types.

### Biotest

In October 2012, approximately 100 L of the RD soils were taken from each nursery, Kle and Alv, at a depth of 0–25 cm from three field replicates. For the treatments, the soils were mixed manually, and one third of the total soil volume per soil type remained either untreated (Con) or was treated at 50°C (H50) or with gamma irradiation (Gamma). The 1-h treatment at 50°C was performed in a dry air oven, using 2 L autoclavable bags. Timing for 1 h was started when the core soil temperature in the bag had reached 50°C which was checked by an inserted thermometer and it took approximately 1 h and half for the two soils used in this study. The soil disinfection with gamma irradiation was applied at a minimal dose of 10 kGy (McNamara et al., 2003) in 15 L autoclavable bags (with no influence of the soil volume and duration). Acclimatized *in vitro* apple rootstock M26 plants, 20 days old, were planted as a susceptible genotype (Kviklys et al., 2008; St. Laurent et al., 2010; Yim et al., 2013), to evaluate the effects of the different RD soil treatments. The experiment was carried out with 10 replicates per treatment in 3 L pots with supplementation of 2 g L^-1^ Osmocote-Exact 3-4M [16 + 9 + 12(+2)], a slow release fertilizer^1^. In total, 60 plants were cultivated in all soil variants (Kle or Alv).

The biotest was set up in a greenhouse during winter time (November 2012) at 20 ± 2°C and a 16 h photoperiod supplied by additional light (Philips Master Agro 400W). The irrigation was applied on a daily basis. Spraying against pests and diseases on aboveground plant parts such as aphids, thrips or spider mites was carried out weekly according to horticultural practices. For data collection the aboveground shoot length (SL) was measured weekly, and after 8 weeks the plants were harvested to determine shoot fresh mass (SFM) and dry mass (SDM) as well as root dry mass (RDM).

For statistical analysis, the homogeneity of variance of SL, SFM, SDM, and RDM was checked prior to the analysis by a Dunnett’s test to check the differences between the control and the treatments. The Tukey test was applied to reveal differences between the three treatments of every measured parameter with *p* < 0.05 using R3.1.0^2^ software.

### Analyses of Soil Bacterial Populations

#### Soil Sampling and Processing

The soil samples used for the bacterial community analyses were collected at the end of the biotest, after the apple M26 plants had been growing for 8 weeks in the greenhouse. Among the 10 replicates per treatment, the soils were taken from eight replicates of the biotest (no. 1–8). Soil attached to roots of the plants was collected by vigorous shaking. Then, soil from two plants was pooled and used as one biological replicate (about 27.2 ± 7.3 g wet soil). In total, 24 soil samples were analyzed (four replicates x three treatments per soil type) after sieving with a mesh size of 2.0 mm. For the DGGE analyses, another eight soil samples taken before the biotest were included (four replicates per soil type).

#### Soil TC-DNA Extraction and Purification

The TC-DNA isolation was accomplished by direct extraction from 500 mg soil from each replicate by bead beating of the FastPrep^®^ Instrument from mpbio (MP Biomedicals, Santa Ana, CA, USA). The extracted nucleic acids were then purified with GENECLEAN SPIN Kit from qbiogene (Qbiogene, Inc., Carlsbad, CA, USA) followed by centrifugal precipitation in 50 μl GENECLEAN^®^ SPIN elution solution according to the protocol described by the manufacturer (MP Biomedicals, Heidelberg, Germany).

#### Amplification of Bacterial 16S rRNA Gene Fragments for Real-Time PCR Analysis

The bacterial 16S rRNA gene copy numbers were quantified using a 5′ Nuclease assay in the real-time quantitative PCR (qPCR). The qPCR reaction mixture (50 μl) consisted of 1x PCR TrueStart^TM^ buffer (Fermentas GmbH, Darmstadt, Germany), 0.2 mM dNTPs, 3 mM MgCl_2_, 5 μg BSA (Bovine Serum Albumin), 1.2 μM BACT1369F as forward primer (5′-CGGTGAATACGTTCYCGG-3′), 1 μM PROK1492R as reverse primer (5′-GGWTACCTTGTTACGACTT-3′), 0.5 μM TM1389F as probe (5′-CTTGTACACACCGCCCGTC-3′), 1.25 U TrueStart^TM^ Taq (Fermentas GmbH, Darmstadt, Germany) and 1 μl TC-DNA (ca. 3 ng). The thermal cycling programs were as previously described by Suzuki et al. (2000).

#### Amplification of Bacterial 16S rRNA Gene Fragments for DGGE Analysis

Amplification of bacterial 16S rRNA gene fragments (GC-PCR) for DGGE fingerprints analysis was carried out as described by Yim et al. (2013), except that 0.5x PCR GoTaq^®^ buffer, 3.75 mM MgCl_2_ and 1.25 U GoTaq^®^ (Promega GmbH, Mannheim, Germany) were used for the PCR reaction (25 μl). The PCR amplification was conducted at 94°C for 5 min, followed by 35 cycles at 94°C for 1 min, 53°C for 1 min, 72°C for 2 min and finally 72°C for 10 min.

#### Amplification of Bacterial 16S rRNA Gene Fragments for 454-Pyrosequencing Analysis

For pyrosequencing of 16S rRNA gene fragments, all TC-DNA samples which had an absorbance ratio A260/A280 between 1.9 and 2.4 (Nanodrop2000c, Spectrophotometer, PEQLAB Biotechnologie GmbH, Erlangen, Germany) were sent for sequencing to the Biotechnology Innovation Center (Roche Life Sciences, BIOCANT, Cantanhede, Portugal). The amplification and sequencing of hypervariable V3-V4 regions of the 16S bacterial ribosomal genes were carried out through the 454 Genome Sequencer FLX platforms according to Roche-Life Sciences using primers 338F (5′-ACTCCTACGGGAGGCAG-3′) and 802R (5′-TACNVRRGTHTCTAATYC-3′) which were fused to the 454 A and B adapters, respectively (Huse et al., 2008; Vaz-Moreira et al., 2011). The PCR reaction mixture (50 μl) contained 5 U of Fast Start Polymerase (Roche Diagnostics, Penzberg, Germany), 3 mM MgCl_2_, 6% DMSO, 0.2 μM of each primer, 200 mM dNTPs and 2 μl of TC-DNA (ca. 3 ng μl^-1^). The PCR conditions were 94°C for 3 min followed by 35 cycles at 94°C for 30 s, 44°C for 45 s, 72°C for 1 min and finally at 72°C for 2 min elongation (Ding et al., 2012).

### Data Analysis

The digital images of silver-stained DGGE gels were analyzed by GelCompar II 6.5 (Applied Math, Sint-Martens-Latern, Belgium). The analysis was based on Pearson correlation coefficients of pairwise similarity measure of two lanes in one gel from the absolute intensity signal in each electrophoresis lane. The UPGMA (unweighted pairwise grouping method using arithmetic means) was applied to obtain a similarity and hierarchical cluster of the lanes. For statistical tests, we used the Pearson similarity matrices from the UPGMA and performed a Permutation test. The test statistics calculated the differences (*d*-value) between the average of all correlation coefficients within the group (within treatment) and the average over all correlation coefficients of different groups (different treatments). Thus, the *d*-value indicated the differences in the bacterial community composition between the soil treatments or soil variants (Kropf et al., 2004).

To check the effect of soil types and of treatments on the bacterial 16S rRNA gene copy numbers by qPCR, ANOVA and Tukey test were applied using R3.1.0^2^ software with *p* < 0.05, respectively.

The analysis of pyrosequencing data was done using Mothur 1.30. software (Schloss et al., 2009). Briefly, the barcode and primer sequences were removed and only those sequences with a length of more than 200 bp were included in the analysis. The trimmed sequences (>200 bp) were aligned to the SILVA 16S rRNA gene database (Pruesse et al., 2007) and the sequence errors were removed by *chimera.uchime*. Classification of sequences into an operational taxonomic unit (OTU) based on 97% sequence similarity for an OTU level report (containing domain, phylum, class, order, family, and genus) according to their taxonomy as well as number of sequences for each of the samples were done as described in Ding et al. (2012). Data were transformed by log(*n*/*N* ^∗^ 100 +1) (*n*, the number of sequences for each OTU and *N*, the total number of sequences from the sample) for the following analyses. The effect of different soil treatments on bacterial relative abundances was checked by the statistical software R3.1.0^3^ using the transformed data and applying Tukey’s honest significant test. Rarefaction analysis was performed to compare the diversity of detected sequences between treatments of both soils based on OTUs defined at 97% similarity. Invsimpson’s diversity index of each sample replicate was used to reveal significant differences of the bacterial diversity between the treatments, applying Tukey test, *p* < 0.05 using R3.1.0.

The principal coordinate analysis (PCoA) using the Bray–Curtis distance metric was carried out with the OTU composition from the dominant phyla (>1% of total sequences in the sample) and only with those OTUs which were identified at the genus level, with Past 3 (3.02). By one-way and two-way ANOSIM tests the differences in the relative abundance of bacterial OTUs between the soil treatments and soil types were tested for significance.

Pyrosequencing data were deposited at the NCBI Sequence Read Archive under the accession number PRJNA276496.

## Results

### Biotest to Reveal Replant Disease Soils

**FIGURE 1:**
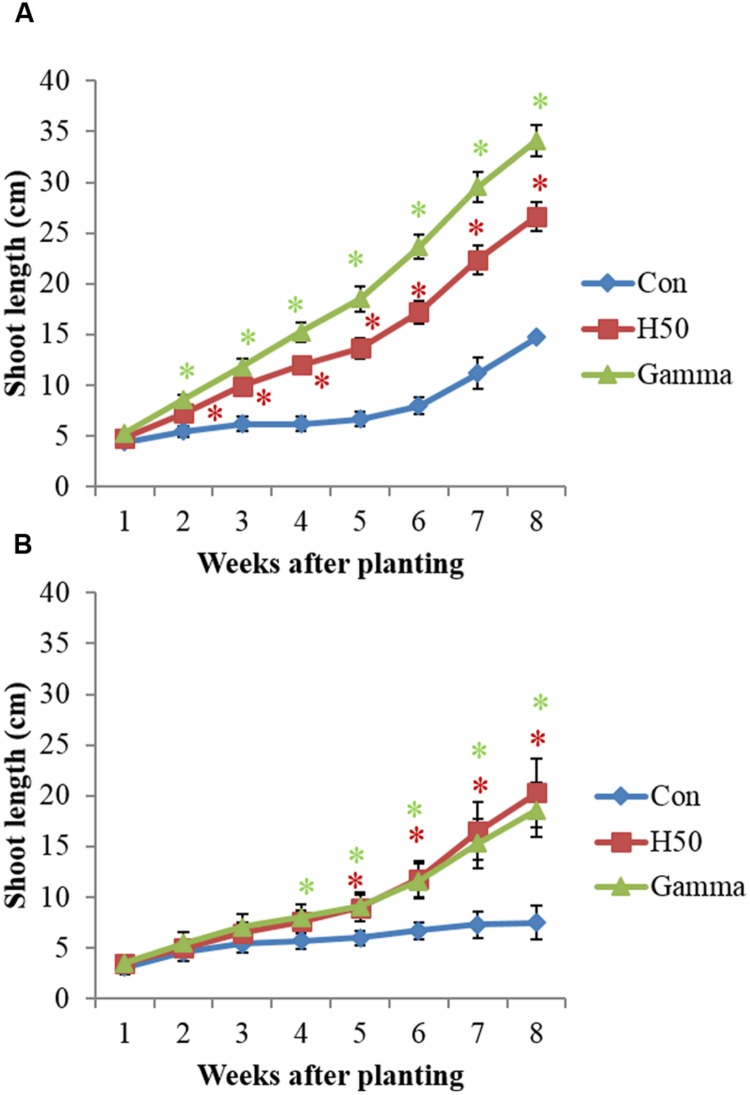
**Growth of apple rootstock M26 plants in Kle **(A)** and Alv **(B)** soil, in different treatments.** Asterisk (^∗^) indicates significant differences between untreated and treated soil (in red, Con vs. H50 and in green, Con vs. Gamma) at the respective time points, Dunnett’s test, *p* < 0.05, I = SD and *n* = 10.

For both RD soils apple M26 plants showed a significantly improved growth in treated (H50, Gamma) compared to untreated (Con) soils (**Figure 1**, **Table 1**). The first significant deviations of SLs between Con and H50/Gamma were noted in Kle soil already 2 weeks after planting (**Figure 1A**). The SL significantly increased by about 81% in H50 soil and by up to 131% in Gamma soil compared to Con soil. In Alv soil, significant deviations of SLs were first recorded 5 weeks after planting (Con vs. H50/Gamma) (**Figure 1B**). In contrast to Kle soil, there was no significant difference for SLs between AlvH50 and AlvGamma (**Figure 1B**). Growth enhancement after gamma irradiation was observed in both soils in comparable extents, since the SLs in AlvGamma was 148% of that in Con soil (**Figure 1**). However, overall growth of M26 plantlets was much higher in Kle than in Alv soil (**Figure 1**).

**Table 1:** Biomass of apple rootstock M26 plants grown for 8 weeks in replant disease (RD) soils after different treatments.

Soil	Treatment	SFM (g/plant)	SDM (g/plant)	% (fold) of increases of SDM	RDM (g/plant)	RDM to SDM ratio
Kle	Con	4.3 ± 1.2^a^	3.1 ± 0.4^a^		2.7 ± 0.6^a^	0.9^a^
	H50	9.2 ± 0.8^b^	4.5 ± 0.3^b^	45 (1.5)	3.0 ± 0.5^a^	0.7^b^
	Gamma	12.7 ± 1.5^c^	5.7 ± 0.4^c^	84 (1.8)	3.1 ± 0.5^a^	0.5^b^

Alv	Con	2.5 ± 1.1^a^	1.5 ± 0.4^a^		3.3 ± 0.1^ab^	2.2^a^
	H50	6.2 ± 2.4^b^	2.6 ± 0.8^b^	73 (1.7)	3.4 ± 0.2^a^	1.3^b^
	Gamma	5.7 ± 2.0^b^	2.3 ± 0.6^b^	53 (1.5)	3.2 ± 0.3^b^	1.4^b^

*Mean ± SD within same parameter and soil followed by different letters indicates significant differences, Tukey test, *p* < 0.05 and *n* = 10. Shoot fresh mass (SFM), shoot dry mass (SDM), and root dry mass (RDM).*

The shoot fresh and dry mass correlated with the SL. The H50 and the Gamma treatment increased the shoot dry mass 1.5- and 1.8-fold, respectively, in Kle soil compared to the control (Con). However, in Alv soil the observed increase of the shoot dry mass was higher in H50 (1.7-fold) than in Gamma-treated soil (1.5-fold). The biomass of roots was not significantly influenced by the treatments in both soils. However, the plants in Con soils showed smaller root systems that were darker brownish in color, and some parts of the roots were necrotic and rotten compared to the roots from H50 and Gamma treatments in both soils (Supplementary Figure S1). In both soils the root-to-shoot ratio was significantly higher in Con soil compared to H50 and Gamma soil (**Table 1**).

### Gene Copy Numbers of 16S rRNA Genes Amplified from Soil TC-DNA

**FIGURE 2:**
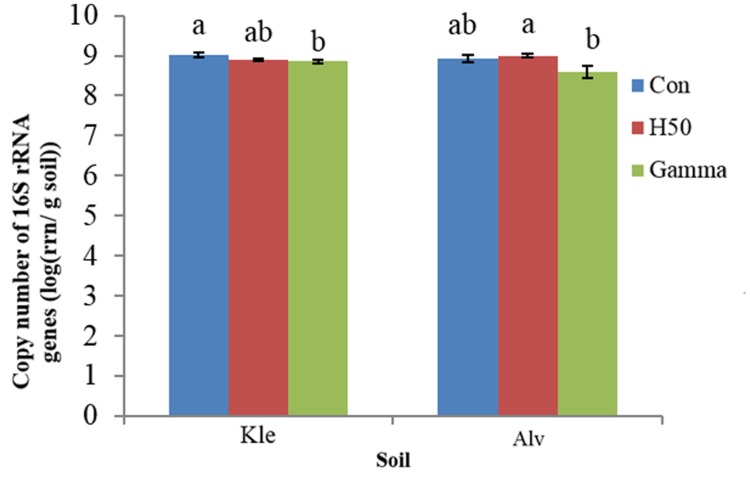
**Copy number of 16S rRNA genes detected in total community DNA of different replant disease (RD) soil treatments, at the end of the biotest.** Letters within soil variant indicate significant differences, Tukey test, *p* < 0.05, *n* = 4 and I = SD.

The qPCR analysis in soil TC-DNA collected 8 weeks after the biotest showed that approximately 10^9^ 16S rRNA gene copy numbers per gram soil were detected with no significant differences in both Con soils (**Figure 2**) (KleCon vs. AlvCon, ANOVA, *p* < 0.05). Only in Kle soil, significantly reduced 16S rRNA gene copy numbers in the Gamma-treated soil were recorded, while the H50 treatment did not influence the numbers in both soils (**Figure 2**).

### DGGE Analysis of 16S rRNA Genes Amplified from Soil TC-DNA

The bacterial DGGE fingerprints of 16S rRNA gene fragments amplified from soil TC-DNA before the biotest revealed significant differences between both soils (KleT0 and AlvT0) which were indicated by dissimilarities in the Permutation test (*d*-value) of 10.6%, *P* = 0.03 (**Table 2**). This demonstrated distinct bacterial community compositions in the two soil types. In both soils, the bacterial community compositions have changed by 5.7 and 5.3% in Kle and Alv soils, respectively, when comparing between sampling time (T0) and at the end of the biotest (Con) after 8 weeks (**Table 2**).

**Table 2:** Treatment-dependent differences of bacterial communities in RD soils before planting and after 8 weeks of the biotest with apple M26 plants (DGGE and pyrosequencing).

Comparison	DGGE		Pyrosequencing	
	*d*-value	*p*-value	*d*-value	*p*-value
KleT0 vs. AlvT0	10.06	0.03	n.a.	n.a.
KleCon vs. KleT0	5.7	0.03	n.a.	n.a.
AlvCon vs. AlvT0	5.3	0.03	n.a.	n.a.
KleCon vs. KleH50	19.2	0.03	22.1	0.01
KleiCon vs. KleGamma	57.6	0.03	29.2	0.02
AlvCon vs. AlvH50	7.6	0.03	7.2	0.04
AlvCon vs. AlvGamma	11.2	0.03	21	0.01

*Percent dissimilarity (*d*-value), average within-group pairwise Pearson’s correlation – average between-group pairwise Pearson’s correlation, *p* < 0.05 and *n* = 4 (Kropf et al., 2004). Abbreviations: T0, samples before planting apple rootstock M26 and Con, H50 and Gamma, samples collected 8 weeks after the biotest (after planting M26); n.a., not analyzed.*

Denaturing gradient gel electrophoresis analysis of 16S rRNA gene fragments amplified from TC-DNA of soil collected at the end of the biotest revealed that the treatments significantly changed the bacterial communities in both soils (**Table 2**). The *d*-values indicated that the H50 treatment resulted in less pronounced shifts in the bacterial community compositions compared to the Gamma treatment, since smaller *d*-values were observed between Con and H50 than between Con and Gamma (**Table 2**).

### Pyrosequencing Analysis of 16S rRNA Genes Amplified from Soil TC-DNA

The pyrosequencing analysis of the V3–V4 region of 16S rRNA gene amplified from soil TC-DNA of samples taken at the end of the biotest resulted in a total of 187,602 sequences with more than 200 bp per sequence from the 24 samples after filtering out low quality or chimeric sequences. All sequences were affiliated to the domain Bacteria. The number of classified sequences ranged between 4,228 and 10,005 sequences per sample, and thus relative abundances were used in the analysis. The sequences were binned based on 97% sequence identity resulting in 10,227 OTUs.

The Permutation test using the pyrosequencing data confirmed the results of the DGGE analyses that after 8 weeks of M26 plant growth the Gamma treatment led to a significantly higher difference of the bacterial community composition compared to Con soil than the H50 treatment (**Table 2**).

**FIGURE 3:**
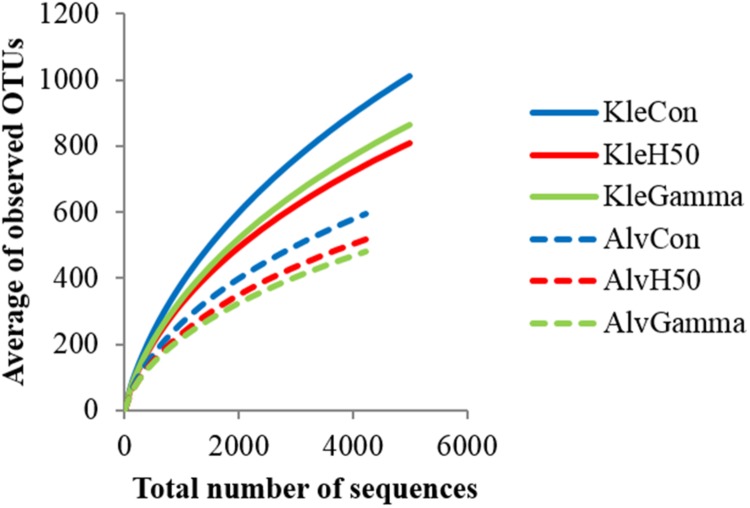
**Rarefaction curves indicating the observed number of operational taxonomic units (OTUs) of bacterial communities.** Diversity of detected sequences, in RD soils, Kle and Alv, 8 weeks after planting apple rootstock M26 plants.

Rarefaction curves allowed a comparison of detected bacterial community diversity with the top curve representing the highest diversity. The bacterial community composition of all treatments of Kle soil was more diverse than that in Alv soil (**Figure 3**). Invsimpson indices of Kle soil (34.9–44.0) were significantly higher than those of Alv soil (11.4–16.3) at *p* < 0.001 (Supplementary Table S4). Within each soil type, the bacterial community diversity in Con soil was by trend higher than in the H50 and Gamma soils, but these differences were not significant (**Figure 3**, Supplementary Table S4).

**FIGURE 4:**
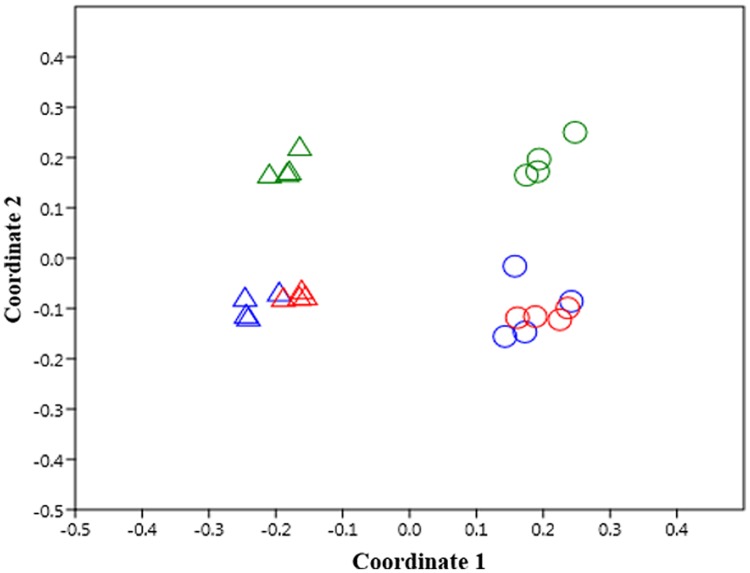
**Effect of soil treatments on the bacterial community composition according to data of operational taxonomic units (OTUs) at genus level as revealed by principal coordinate analysis using the Bray–Curtis distance metric, *n* = 4.** Δ and ◯ are for Kle and Alv soils, respectively. Colors in blue, red and green represent Con, H50 and Gamma treatment, respectively.

Principal coordinate analysis considering the bacterial community composition at the genus level clearly separated Kle soil from Alv soil (**Figure 4**) (ANOSIM test, *R* = 0.94, *p* < 0.001). For both Kle and Alv soils, differences were also recorded between Con and H50 soil, but a more pronounced dissimilarity was observed for Gamma and Con as well as for Gamma and H50 soils (**Figure 4**). Overall, the ANOSIM tests showed that after soil treatments the bacterial community composition significantly shifted for both soils (*R*-values of 1.0 and 0.77 for Kle and Alv soils, respectively, and *p* < 0.001).

### Taxonomic Composition of Bacteria in Replant Disease Soils

**FIGURE 5:**
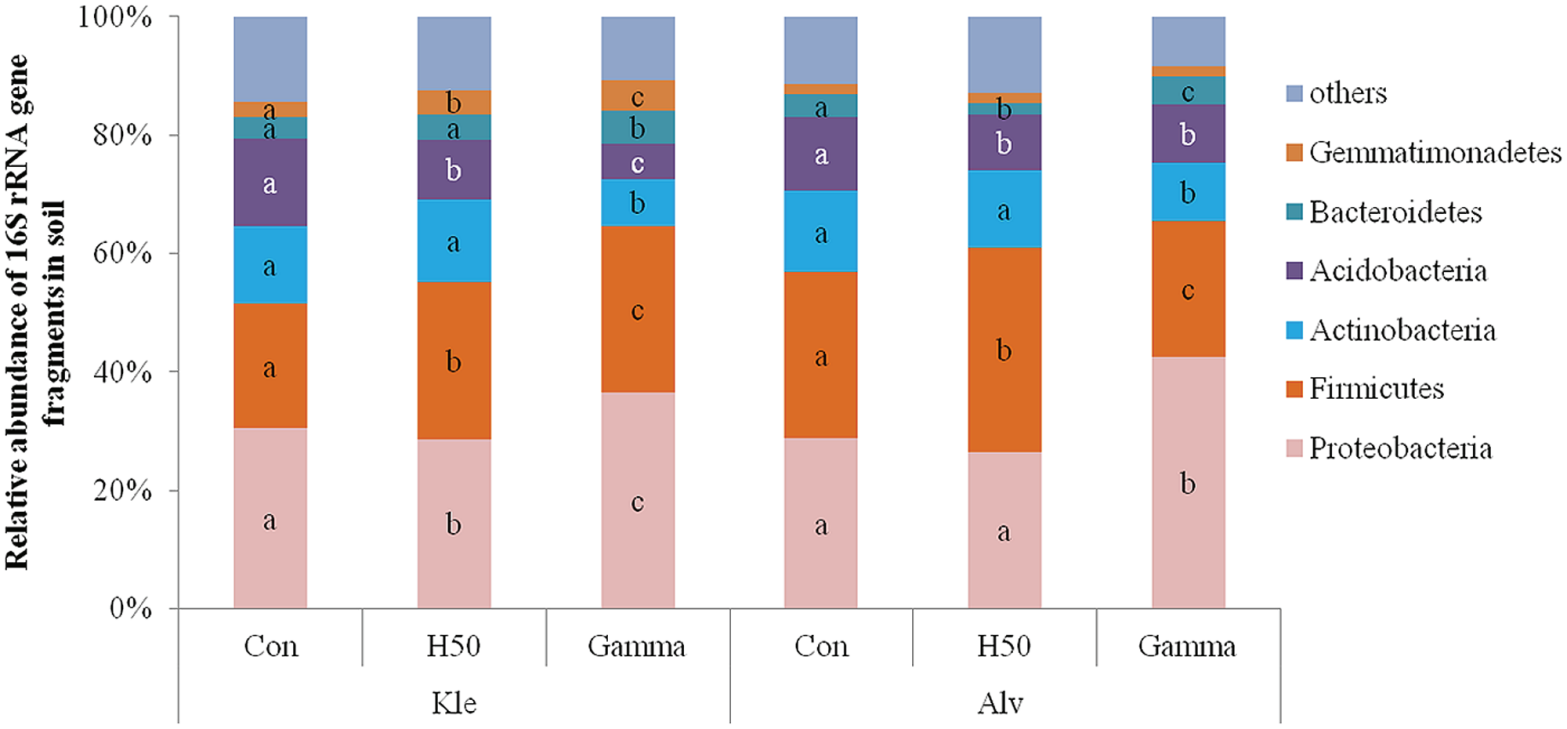
**Relative abundance of dominant phyla detected in RD soils at the end of the biotest affected by different treatments.** Different letters within phylum and within soil type indicated significant differences between treatments, Tukey test, *p* < 0.05 and *n* = 4.

A total of 10,227 OTUs from 22 phyla, 62 classes, 98 orders, 185 families, and 342 genera were identified in both soils (Kle and Alv). Phyla with a relative abundance below 1% were considered rare (Supplementary Table S3 shows relative abundance of 13 phyla). The dominant phyla were *Proteobacteria, Firmicutes, Acidobacteria, Actinobacteria, Bacteroidetes*, and *Gemmatimonadetes* (**Figure 5**) to which 96.3 and 96% of the sequences in Kle and Alv soils were affiliated, respectively. An average of 30.5 and 28.8% of the total sequences for Kle and Alv soils, respectively, were assigned to the *Proteobacteria* (**Figure 5**). Within the phylum *Proteobacteria, Alphaproteobacteria* were the most abundant followed by *Gammaproteobacteria, Betaproteobacteria*, and *Deltaproteobacteria*.

Although the gene copy numbers of 16S rRNA detected in TC-DNA of RD soils were comparable (KleCon vs. AlvCon) (**Figure 2**), the bacterial community composition differed (Supplementary Table S2). For instance, the relative abundance of *Acidobacteria* and *Gemmatimonadetes* was significantly lower in AlvCon compared to KleCon. At the genus level, members of the genera *Gp1, Gp2, Gp3, Gp6, Gp16, Arthrobacter, Bacillus, Paenibacillus, Clostridium sensu stricto, Gemmatimonas*, and *Sphingomonas* were dominant in both Con soils (**Table 3**). Many genera such as *Gp1, Gp2, Arthrobacter, Nocardioides, Paenibacillus, Phenylobacterium, Lysobacter*, and others (Supplementary Table S2) had a similar relative abundance in both soils. Other genera such as *Bacillus* were significantly higher in relative abundance in AlvCon soil (11.1 ± 4%) than in KleCon soil (Supplementary Table S2).

**Table 3:** Relative abundance of dominant genera detected at the end of the biotest in RD soils of Kle and Alv affected by soil treatments.

Phylum	Genus	Kle			Alv		
		Con	H50	Gamma	Con	H50	Gamma
*Acidobacteria*	*Gp1*	3.9 ± 0^a^	3.5 ± 0^a^	1.7 ± 0^b^	3.4 ± 1	3.3 ± 1	2.9 ± 1
	*Gp2*	1.8 ± 0^a^	0.9 ± 0^b^	0.4 ± 0^c^	1.8 ± 1^a^	0.6 ± 0^b^	0.7 ± 0^b^
	*Gp3*	3.7 ± 0^a^	2.2 ± 0^b^	1.2 ± 0^c^	3 ± 1^a^	2.1 ± 1^b^	1.3 ± 0^c^
	*Gp4*	0.8 ± 0^a^	0.6 ± 0^ab^	0.4 ± 0^b^	0.1 ± 0	0.2 ± 0	0 ± 0
	*Gp5*	0.3 ± 0^a^	0 ± 0^b^	0.1 ± 0^b^	0.1 ± 0	0.1 ± 0	0 ± 0
	*Gp6*	1.1 ± 0^a^	0.4 ± 0^b^	0.5 ± 0^b^	0.3 ± 0	0.2 ± 0	0.2 ± 0
	*Gp7*	0.5 ± 0^a^	0.3 ± 0^b^	0.1 ± 0^b^	0.2 ± 0	0.2 ± 0	0.2 ± 0
	*Gp13*	0.1 ± 0^a^	0 ± 0^b^	0 ± 0^b^	0.1 ± 0	0 ± 0	0 ± 0
	*Gp14*	0 ± 0^a^	0.2 ± 0^b^	0.1 ± 0^ab^	0.5 ± 0	0.5 ± 0	0.4 ± 0
	*Gp16*	1.6 ± 0^a^	1.4 ± 0^a^	0.5 ± 0^b^	0.8 ± 0^a^	0.6 ± 0^a^	0.3 ± 0^b^
	*Granulicella*	0 ± 0	0 ± 0	0 ± 0	0.1 ± 0^a^	0 ± 0^a^	0.4 ± 1^b^

*Actinobacteria*	*Ilumatobacter*	0.4 ± 0^a^	0.1 ± 0^b^	0 ± 0^b^	0.1 ± 0	0 ± 0	0 ± 0
	*Blastococcus*	0.2 ± 0^a^	0.5 ± 0^b^	0.1 ± 0^a^	0.1 ± 0^ab^	0.2 ± 0^a^	0 ± 0^b^
	*Arthrobacter*	1.1 ± 0	0.8 ± 0	0.9 ± 0	0.9 ± 1^a^	1 ± 1^a^	1.8 ± 0^b^
	*Mycobacterium*	0 ± 0	0.1 ± 0	0 ± 0	0.3 ± 0^a^	0.2 ± 0^ab^	0.1 ± 0^b^
	*Nocardioides*	0.9 ± 0	0.8 ± 0	0.8 ± 0	0.7 ± 0^a^	0.6 ± 0^a^	0.2 ± 0^b^
	*Pseudonocardia*	0.2 ± 0	0.1 ± 0	0.1 ± 0	0.3 ± 0^a^	0.1 ± 0^b^	0.1 ± 0^b^
	*Streptomyces*	0.1 ± 0^a^	0.6 ± 0^b^	0.2 ± 0^a^	0.2 ± 0^a^	0.5 ± 0^b^	0.2 ± 0^a^

*Bacteroidetes*	*Niastella*	0 ± 0^a^	0.3 ± 0^b^	0 ± 0^a^	0 ± 0	0 ± 0	0 ± 0
	*Mucilaginibacter*	0.1 ± 0^a^	0.1 ± 0^a^	0.3 ± 0^b^	0.1 ± 0^a^	0 ± 0^a^	0.5 ± 0^b^

*Firmicutes*	*Bacillus*	4.1 ± 1^a^	5.1 ± 0^b^	5.5 ± 0^b^	11.1 ± 4^a^	13.2 ± 3^b^	7.6 ± 2^c^
	*Tuberibacillus*	0.2 ± 0^a^	0 ± 0^b^	0 ± 0^b^	0 ± 0	0 ± 0	0 ± 0
	*Brevibacillus*	0.1 ± 0^a^	0.4 ± 0^b^	0.1 ± 0^a^	0.1 ± 0	0.1 ± 0	0.1 ± 0
	*Paenibacillus*	1.4 ± 0^a^	2.1 ± 0^b^	1.6 ± 0^a^	1.4 ± 0^a^	2.5 ± 1^b^	1.7 ± 1^a^
	*Clostridium sensu stricto*	0.9 ± 0	0.9 ± 0	1 ± 0	2 ± 1^a^	2.4 ± 1^a^	0.7 ± 0^b^
	*Clostridium XI*	0.6 ± 0^a^	0.5 ± 0^a^	1 ± 0^b^	0.5 ± 0	0.6 ± 0	0.5 ± 0
	*Clostridium III*	0.3 ± 0	0.3 ± 0	0.5 ± 0	0.3 ± 0^a^	0.3 ± 0^a^	0.1 ± 0^b^

*Gemmatimonadetes*	*Gemmatimonas*	2.6 ± 0^a^	4.1 ± 1^b^	5.2 ± 1^c^	1.7 ± 0	1.8 ± 1	1.7 ± 1

*Proteobacteria (Alpha)*	*Phenylobacterium*	0.4 ± 0^a^	1.1 ± 0^b^	0.6 ± 0^c^	0.3 ± 0^a^	0.6 ± 0^b^	0.6 ± 0^b^
	*Devosia*	0.3 ± 0^a^	0.2 ± 0^a^	0.8 ± 0^b^	0.3 ± 0^a^	0.1 ± 0^a^	0.8 ± 0^b^
	*Microvirga*	0 ± 0^a^	0.2 ± 0^b^	1.3 ± 0^c^	0 ± 0	0.1 ± 0	0 ± 0
	*Mesorhizobium*	0.3 ± 0^a^	0.1 ± 0^b^	0.5 ± 0^a^	0.1 ± 0^ab^	0 ± 0^a^	0.3 ± 0^b^
	*Acidocella*	0 ± 0	0 ± 0	0 ± 0	0.6 ± 0^a^	0.1 ± 0^b^	0.9 ± 1^a^
	*Sphingomonas*	1.7 ± 0^a^	2.7 ± 0^b^	1.8 ± 0^a^	0.7 ± 1^a^	2.6 ± 1^b^	1.7 ± 0^c^

*Proteobacteria (Beta)*	*Burkholderia*	0.1 ± 0^a^	0.6 ± 0^b^	1.3 ± 0^c^	0.5 ± 0	0.8 ± 0	0.8 ± 0
	*Ramlibacter*	0 ± 0^a^	0.2 ± 0^b^	0.4 ± 0^c^	0 ± 0	0 ± 0	0.1 ± 0

*Proteobacteria (Delta)*	*Geobacter*	0.2 ± 0^a^	0 ± 0^b^	0.2 ± 0^a^	0.1 ± 0	0.1 ± 0	0 ± 0

*Proteobacteria (Gamma)*	*Methylobacter*	0.2 ± 0	0.1 ± 0	0.1 ± 0	0.2 ± 0^a^	0.1 ± 0^b^	0 ± 0^b^
	*Arenimonas*	0.1 ± 0^a^	0 ± 0^a^	0.6 ± 0^b^	0 ± 0	0 ± 0	0 ± 0
	*Dokdonella*	0.5 ± 0^a^	0.2 ± 0^b^	0.1 ± 0^b^	0.4 ± 0^a^	0.1 ± 0^b^	0.1 ± 0^b^
	*Dyella*	0.2 ± 0^a^	0 ± 0^b^	0.2 ± 0^a^	0.2 ± 0	0.2 ± 0	0 ± 0
	*Lysobacter*	0.2 ± 0^a^	0.3 ± 0^ab^	0.4 ± 0^b^	0.1 ± 0^ab^	0 ± 0^a^	0.2 ± 0^b^
	*Rhodanobacter*	0.2 ± 0^a^	0.2 ± 0^a^	1 ± 0^b^	0.8 ± 1^a^	0.8 ± 0^a^	3.6 ± 1^b^

*Selected were genera of a relative abundance of ≥0.1% and significantly different within the soil type. Average relative abundances ± SD, significant differences between treatments within soil type at genus level were indicated by different letters after Tukey test, *p* < 0.05 and *n* = 4. Significant increases in abundance compared to Con are highlighted in green, while significant decreases are highlighted in orange.*

### Treatment-Dependent Bacterial Responders

Even 8 weeks after apple rootstock growth, changes in the relative abundances of different phyla were recorded in the treated soils compared to the control. In H50 soil, a significant decrease in the relative abundance of the phylum *Acidobacteria* and an increase of the phylum *Firmicutes* was observed in both soils. In Gamma soil the relative abundances of the phyla *Actinobacteria* and *Acidobacteria* were significantly reduced, whereas the relative abundances of the *Proteobacteria* and the *Bacteroidetes* were significantly increased in both soils (**Figure 5**).

Genera with significantly higher or lower relative abundance in response to the treatment (so-called responders) were different in both soils. In Kle soil, both treatments (H50, Gamma) reduced the relative abundances of several genera belonging to the *Acidobacteria* (*Gp2, Gp3, Gp5, Gp6, Gp7*, and *Gp13*) as well as the genera *Ilumatobacter, Tuberibacillus*, and *Dokdonella*. The decrease in relative abundances of the acidobacterial genera was less pronounced in Alv soil and only *Gp2, Gp3*, and *Dokdonella* were significantly less abundant (H50, Gamma). *Pseudonocardia* and *Methylobacter* showed a significantly decreased relative abundance in both H50 and Gamma treatments of Alv soil, while a significantly decreased relative abundance of the genera *Mycobacterium, Nocardioides, Bacillus, Clostridium sensu stricto*, and *Clostridium III* were observed only in AlvGamma soil (**Table 3**).

Soil treatments and the M26 plants not only decreased, but also enriched the relative abundances of a wide range of different genera compared to the control soils. Significantly increased abundances in both KleH50 and KleGamma compared to Con soil were observed for the genera *Bacillus, Gemmatimonas, Phenylobacterium, Microvirga, Burkholderia* and *Ramlibacter*. In Alv soil the genera *Streptomyces* and *Paenibacillus* significantly increased in abundance in H50 soil, while *Granulicella, Arthrobacter, Mucilaginibacter, Devosia*, and *Rhodanobacter* showed a significantly higher relative abundance in Gamma soil. Besides soil type specific treatment responses, only a few genera were recorded as responders to the treatment in both soils. Remarkably, *Streptomyces, Bacillus, Paenibacillus*, and *Sphingomonas* showed a significantly increased abundance in the H50 soil of Alv and Kle soils, while *Mucilaginibacter, Devosia*, and *Rhodanobacter* were detected in higher relative abundance in the Gamma treatment of the two soils. Very few genera even showed the same response to the H50 and Gamma treatments in both soil types: while *Gp2, Gp3*, and *Dokdonella* showed a decrease in relative abundance, a significantly increased abundance was observed for the genus *Phenylobacterium*.

## Discussion

### Biotest

Growth of apple rootstock M26 plants improved significantly in RD soils after H50 or Gamma treatments (**Table 1**). The enhanced plant growth was mainly observed aboveground while the treatments did not affect RDM. Significant increases of the shoot growth and biomass of apple M26 plants in heat-treated soil were also observed in the studies by St. Laurent et al. (2010) and Yim et al. (2013). The differences in growth of M26 plants in both RD soils were associated with differences in soil physicochemical properties and cropping histories. Among other functions, roots are important for water and nutrient uptake, release of exudates and production of cytokinins for the shoot growth (Gregory, 2006). Although there were no significant differences in the RDM of apple M26 plants in different RD soil treatments, damages in the root system of the plants in Con soils have resulted in higher root-to-shoot ratios (**Table 1**, Supplementary Figure S1). Since the roots were damaged in RD soil (Con), the plants might have invested energy in defense reactions of the root. Similarly, in the study of Yim et al. (2013), histological analyses of apple roots grown in RD soil revealed more lignin in vascular cells and a secondary protecting layer derived from the endodermis. The stronger lignifications might have resulted from oxidation of phenolic compounds that are known to play an important role in plant defense mechanisms. Several reviews have reported that under stress conditions biosynthesis of antimicrobial metabolites was enhanced as a defense mechanism of the plant (Sticher et al., 1997; Doornbos et al., 2012; Badri et al., 2013). The brownish roots of M26 grown in RD soil (Supplementary Figure S1) could have resulted from such a stress response of the plants. Phytochemicals were contained in, and released from roots in high quantities in response to biotic stress (Badri et al., 2013). Hofmann et al. (2009) have identified phloridzin (phloretin-2-β-D-glucoside) as the most abundant phenolic root exudate detected in apple seedlings (*Malus x domestica* Borkh.). Likewise, Emmett et al. (2014) reported that the production of phloridzin in roots of apple rootstock M26 plants in untreated RD soil was significantly higher than in pasteurized RD soil. Chizzali et al. (2012) detected phytoalexins including the biphenyls 3-hydroxy-5-methoxyaucuparin, aucuparin and others in the transition zone of apple stems as a result of plant defense responses against fire blight caused by *Erwinia amylovora*.

The increased biosynthesis of phenylpropanoids in young apple leaves was shown to be negatively correlated with the shoot growth (Rühmann et al., 2002). Thus, we hypothesize that an inverse relationship between shoot growth and biosynthesis of phenolic compounds or antimicrobial metabolites could be the explanation for the reduced biomass of the apple M26 plants in RD soil observed in the present study.

### Soil Bacterial Composition and Diversity in RD Soils

Although the copy numbers of 16S rRNA genes detected in TC-DNA of both RD soils (KleCon/AlvCon) at the end of the biotest revealed no differences (**Figure 2**), distinct bacterial community compositions (**Figure 4**) and diversity (**Figure 3**) were recorded in the present study (Supplementary Table S2). Differences in the bacterial community compositions were also shown in the two soils collected before the biotest (**Table 2**). Several other studies had shown that soil bacterial communities were strongly correlated to soil physicochemical properties (Janssen, 2006; Araujo et al., 2012; Schreiter et al., 2014). The soils used in the present study differed in their mineral composition, pH, organic matter content, cropping histories and horticultural management. Rose and apple rootstock plants were previously cultivated in Kle and Alv soils, respectively, and crop rotation was applied mainly in Kle soil. Plant species and soil type dependent diversity of bacterial communities was shown in different studies (Smalla et al., 2001; Badri et al., 2013; Bulgarelli et al., 2013) and thus the crop rotation might have contributed to the higher bacterial diversity found in Kle soil.

The relative abundance of common responders in both RD soils most likely was influenced by plant root exudates released by the apple rootstocks cultivated in these soils in 2012 (M4 planted in May. and M26 in November 2012), as also shown for other crops (Smalla et al., 2001; Berg and Smalla, 2009; Bakker et al., 2012; Berendsen et al., 2012). Soil type-dependent differences of the root exudate composition for the same plant species (lettuce) grown in different soil types were recently reported by Neumann et al. (2014). Apple rootstock exudates might have influenced the bacterial community composition contributing to the differences observed between the DGGE fingerprints of the RD soils before and after the biotest (KleT0 vs. KleCon, AlvT0 vs. AlvCon, **Table 2**).

Sun et al. (2014) have also studied the bacterial diversity associated with RD soils in apple orchards. Only a few genera such as *Lysobacter* and *Phenylobacterium* detected by Sun et al. (2014) were also identified in the present study. The relative abundances of the genera *Lysobacter* and *Phenylobacterium* were higher in RD soil than in healthy soil (Sun et al., 2014). In our experimental design healthy soil was not included as it was difficult to obtain soil with similar chemical and physical properties. In contrast, in the present study the relative abundance of the genus *Phenylobacterium* was significantly higher in RD soil with H50 and with Gamma treatment in both soils, while *Lysobacter* was enriched in the Gamma-treated Kle soil (**Table 3**).

Based on plant, soil, and soil bacterial community interaction, we hypothesized that soil bacterial community composition and diversity are site specific, influenced by different chemical and physical properties of the soil, as well as shaped by planting management practices. Also in other systems it was shown that the microbial community composition and the abundance of soilborne pathogens are influenced by the soil type, cropping history and weather conditions (Smalla et al., 2001; Badri and Vivanco, 2009; Berg and Smalla, 2009; Bakker et al., 2012; Berendsen et al., 2012; Badri et al., 2013).

### Responses of the Bacterial Composition and Diversity in RD Soils to Different Treatments

The 16S rRNA gene copy numbers detected in soil TC-DNA showed a minor but still significant reduction only in Gamma-treated Kle soil 8 weeks after planting apple rootstock M26 plants (Con vs. Gamma) (**Figure 2**). Thus, recolonization of the soil must have taken place within this time span which was most likely influenced by the growing apple rootstock. The integration of an unplanted control would have allowed elucidating the effect of the plant growth and should be included in future experiments. In the study by McNamara et al. (2007) the bacterial counts decreased immediately after irradiation at a dose of 10 kGy, but 2 weeks later the cell counts rose to levels of up to 10^7^ g^-1^ soil, which was even higher than in the untreated soil (10^6^ g^-1^). The soil analyzed in the present study loosely adhered to the root; a stronger influence of the plant would be expected if true rhizosphere soil was analyzed.

Both treatments of RD soils caused pronounced shifts in the bacterial community composition compared to the control which were detectable even 8 weeks after apple rootstock growth, with the effect of the Gamma treatment being more pronounced (**Figure 4**). Although the response of the acidobacterial populations was more striking in Kle soil, a decrease in the relative abundance of *Acidobacteria* was observed in response to the treatments in both soils (**Figure 5**). A significantly decreased relative abundance of the phylum *Acidobacteria* after treatment of maize RD soil with ethanol-free chloroform was recently reported by Domínguez-Mendoza et al. (2014). *Acidobacteria* were detected in apple RD soil as the dominant phylum, and their abundance was shown to be about 20% higher in soils in which the apple rootstock genotype M26 (considered susceptible to RD) was cultivated than in soils where the more tolerant genotype CG6210 was grown (St. Laurent et al., 2010). A significantly decreased relative abundance of *Acidobacteria* was also reported when treating soil with manure (Ding et al., 2014) or with mineral nutrients (Campbell et al., 2010). Therefore, the decreased relative abundance of *Acidobacteria* in treated RD soil observed in the present study might result from the release of nutrients from killed organisms due to the treatments and the proliferation of copiotrophic bacteria.

The genera *Nocardioides, Clostridium sensu strictu*, and *Clostridium III* were significantly reduced in Gamma-treated Alv soil (**Table 3**). *Nocardioides* also significantly decreased in relative abundance by soil sterilization with ethanol-free chloroform in maize RD soil (Domínguez-Mendoza et al., 2014). Isolates belonging to the genus *Nocardioides* were reported as beneficial bacteria as they contributed to carbon cycling in soil via degrading alkanes (Hamamura et al., 2001) and via degrading pesticides (Topp et al., 2000).

In the present study, soil treatments did not only reduce but also strongly enrich the relative abundances of a wide range of bacterial genera. *Bacillus, Paenibacillus*, and *Sphingomonas* were shown to increase in relative abundance with H50 treatment of both soils (**Table 3**). Isolates belonging to the genera *Bacillus* and *Paenibacillus* were reported to be involved in the early stage of mineralization of decomposable organic materials derived from killed soil microorganisms (Domínguez-Mendoza et al., 2014). Bruce et al. (2010) have shown that most of the isolates from soil belonging to the genera *Bacillus* and *Paenibacillus* play a role in carbon cycling as they degrade cellulose and lignin. The genus *Bacillus* is known to contain plant growth promoting bacteria with most of the isolates being able to produce indole acetic acid (IAA), ammonia, siderophores, catalase (Joseph et al., 2007) and antibiotics against soilborne pathogenic fungi (Cazorla et al., 2007). Antagonistic activity of several *Sphingomonas* isolates from plants against pathogenic *Pseudomonas syringae* in *Arabidopsis thaliana* was revealed by Innerebner et al. (2011).

Members of the genera *Mucilaginibacter, Devosia*, and *Rhodanobacter* showed significantly increased relative abundance only in the Gamma treatments of both soils (**Table 3**). *Mucilaginibacter* species are heterotrophic bacteria capable to degrade pectin, xylan, laminarin and other polysaccharides (Pankratov et al., 2007). Isolates from the genus *Devosia* were also reported as plant growth promoting bacteria, e.g., *D. neptuniae* is capable to fix nitrogen in the roots of the aquatic legume plant *Neptunianatans* (Rivas et al., 2002). Isolates of the genus *Rhodanobacter* from subsurface area contaminated with uranium and nitric acid wastes were identified as denitrifying bacteria (Green et al., 2010).

The treatment-dependent enrichment of potentially beneficial or aromatic compound degrading bacteria in treated RD soil might have contributed to the enhanced growth of apple rootstock M26 plants in treated RD soils.

## Conclusion

Apple rootstock M26 plants showed significant growth enhancement in treated RD soil after heat treatment at 50°C or gamma irradiation in a biotest. The DGGE and pyrosequencing analyses of 16S rRNA gene fragments amplified from TC-DNA of soil collected from M26 plant roots at the end of the biotest revealed distinct bacterial community compositions and diversity between the two RD soils. The pronounced differences in the relative abundance of soil bacteria were affected directly by soil treatments, by recolonization and proliferation after treatment, and by the plant root exudates. The 16S rRNA gene-based approaches can indicate changes in the relative abundance in response to treatments that might have contributed to the improved aboveground growth. However, conclusions concerning the potential activity and role of responders remain purely speculative. Thus, a polyphasic approach is urgently needed to shed more light on the phenomenon of RDs.

## Conflict of Interest Statement

The authors declare that the research was conducted in the absence of any commercial or financial relationships that could be construed as a potential conflict of interest.

## Footnotes

1 http://www.scottsprofessional.com

2 http://www.r-project.org

3 http://www.r-project.org
