# Supplementary material for: Different bacterial communities in heat and gamma irradiation treated replant disease soils revealed by 16S rRNA gene analysis – contribution to improved aboveground apple plant growth?
Source: Front Microbiol. 2015 Nov 6;6:1224. doi: 10.3389/fmicb.2015.01224 (PMC4654428; doi:10.3389/fmicb.2015.01224)
Supplement: Supplementary file 1 [file Data_Sheet_1.DOCX]

**S1 Table. Characterization of the soils used in the study.**

| **Soil** | **Soil type** | **pH** | **Mg**  **(mg/100g)** | **P_2_O_5_**  **(mg/100g)** | **K_2_O**  **(mg/100g)** | **Organic matter (%)** | **Clay (%)** | **Silt (%)** | **Sand (%)** |
| --- | --- | --- | --- | --- | --- | --- | --- | --- | --- |
| **Kle** | **Sandy soil** | 5.2 | 4.8 | 23.4 | 11.9 | 4.2 | 3 | 4 | 93 |
| **Alv** | **Slightly loamy sand** | 4.8 | 2.7 | 22.7 | 13.2 | 3.7 | 7 | 14 | 79 |

Method of soil fraction analysis (Van Reeuwijk, 2002), of pH (Methodenbuch VDLUFA Bd. I, A 5.1.1, 1991-06) and of nutrient analysis (CAL, VDLUFA-Methodenbuch Bd.1, A 6.2.1.1.1 1991-06).

**S2 Table. Relative abundances of bacterial composition and diversity detected eight weeks after planting apple rootstock M26 in two replant disease soils.**

| **Phyla** | **Class** | **Genus** | **KleCon** | **AlvCon** |
| --- | --- | --- | --- | --- |
| ***Acidobacteria*** |  |  | 14.7±0 | 12.4±2* |
|  | *Acidobacteria_Gp1* | *Gp1* | 3.9±0 | 3.4±1 |
|  | *Acidobacteria_Gp2* | *Gp2* | 1.8±0 | 1.8±1 |
|  | *Acidobacteria_Gp3* | *Gp3* | 3.7±0 | 3±1* |
|  | *Acidobacteria_Gp4* | *Gp4* | 0.8±0 | 0.1±0* |
|  | *Acidobacteria_Gp5* | *Gp5* | 0.3±0 | 0.1±0* |
|  | *Acidobacteria_Gp6* | *Gp6* | 1.1±0 | 0.3±0* |
|  | *Acidobacteria_Gp7* | *Gp7* | 0.5±0 | 0.2±0* |
|  | *Acidobacteria_Gp13* | *Gp13* | 0.1±0 | 0.1±0 |
|  | *Acidobacteria_Gp14* | *Gp14* | 0±0 | 0.5±0* |
|  | *Acidobacteria_Gp16* | *Gp16* | 1.6±0 | 0.8±0* |
| ***Actinobacteria*** |  |  | 13±1 | 13.9±1 |
|  | *Actinobacteria* | *Ilumatobacter* | 0.4±0 | 0.1±0* |
|  | *Actinobacteria* | *Blastococcus* | 0.2±0 | 0.1±0 |
|  | *Actinobacteria* | *Arthrobacter* | 1.1±0 | 0.9±1 |
|  | *Actinobacteria* | *Mycobacterium* | 0±0 | 0.3±0* |
|  | *Actinobacteria* | *Nocardioides* | 0.9±0 | 0.7±0 |
|  | *Actinobacteria* | *Pseudonocardia* | 0.2±0 | 0.3±0 |
|  | *Actinobacteria* | *Streptomyces* | 0.1±0 | 0.2±0 |
| ***Bacteroidetes*** |  |  | 3.8±0 | 3.7±2 |
|  | *Bacteroidetes_incertae_sedis* | *Ohtaekwangia* | 0.1±0 | 0.1±0 |
|  | *Sphingobacteria* | *Mucilaginibacter* | 0.1±0 | 0.1±0 |
|  | *Sphingobacteria* | *Pedobacter* | 0.1±0 | 0±0 |
| ***Firmicutes*** |  |  | 21.1±3 | 28±11 |
|  | *Bacilli* | *Bacillus* | 4.1±1 | 11.1±4* |
|  | *Bacilli* | *Tuberibacillus* | 0.2±0 | 0±0 |
|  | *Bacilli* | *Brevibacillus* | 0.1±0 | 0.1±0 |
|  | *Bacilli* | *Paenibacillus* | 1.4±0 | 1.4±0 |
|  | *Bacilli* | *Ureibacillus* | 0.1±0 | 0±0 |
|  | *Bacilli* | *Thermoactinomyces* | 0.4±0 | 0.1±0* |
|  | *Bacilli* | *Planifilum* | 0.5±0 | 0±0* |
|  | *Clostridia* | *Clostridium sensu stricto* | 0.9±0 | 2±1* |
|  | *Clostridia* | *Symbiobacterium* | 0.1±0 | 0±0 |
|  | *Clostridia* | *Clostridium XI* | 0.6±0 | 0.5±0 |
|  | *Clostridia* | *Clostridium III* | 0.3±0 | 0.3±0 |
|  | *Erysipelotrichia* | *Turicibacter* | 0.4±0 | 0.2±0* |
|  | *Negativicutes* | *Psychrosinus* | 0.1±0 | 0.3±0* |
| ***Gemmatimonadetes*** |  |  | 2.6±0 | 1.7±0* |
|  | *Gemmatimonadetes* | *Gemmatimonas* | 2.6±0 | 1.7±0* |
| ***Proteobacteria*** |  |  | 30.5±2 | 28.8±7 |
|  | ***Alphaproteobacteria*** |  | 18.3±2 | 14.6±5* |
|  | *Alphaproteobacteria* | *Phenylobacterium* | 0.4±0 | 0.3±0 |
|  | *Alphaproteobacteria* | *Devosia* | 0.3±0 | 0.3±0 |
|  | *Alphaproteobacteria* | *Mesorhizobium* | 0.3±0 | 0.1±0* |
|  | *Alphaproteobacteria* | *Acidocella* | 0±0 | 0.6±0* |
|  | *Alphaproteobacteria* | *Novosphingobium* | 0.4±0 | 0.3±1 |
|  | *Alphaproteobacteria* | *Sphingomonas* | 1.7±0 | 0.7±1* |
|  | ***Betaproteobacteria*** |  | 4.5±0 | 4.5±1 |
|  | *Betaproteobacteria* | *Burkholderia* | 0.1±0 | 0.5±0* |
|  | *Betaproteobacteria* | *Nitrosospira* | 0.2±0 | 0.2±0 |
|  | ***Deltaproteobacteria*** |  | 2.5±0 | 2±0 |
|  | *Deltaproteobacteria* | *Geobacter* | 0.2±0 | 0.1±0 |
|  | ***Gammaproteobacteria*** |  | 4.2±0 | 6.8±1* |
|  | *Gammaproteobacteria* | *Aquicella* | 0.2±0 | 0.1±0 |
|  | *Gammaproteobacteria* | *Methylobacter* | 0.2±0 | 0.2±0 |
|  | *Gammaproteobacteria* | *Arenimonas* | 0.1±0 | 0±0 |
|  | *Gammaproteobacteria* | *Dokdonella* | 0.5±0 | 0.4±0 |
|  | *Gammaproteobacteria* | *Dyella* | 0.2±0 | 0.2±0 |
|  | *Gammaproteobacteria* | *Lysobacter* | 0.2±0 | 0.1±0 |
|  | *Gammaproteobacteria* | *Rhodanobacter* | 0.2±0 | 0.8±1* |

(Average relative abundances ±SD), significant differences in relative abundances between soil types at genus level were indicated by asterisk "*", ANOVA, p < 0.05 and n = 4. Significant increases in relative abundances are highlighted in green, while significant decreases are highlighted in orange.

**S3 Table. Relative abundances of bacterial composition and diversity detected eight weeks after planting apple rootstock M26 in replant disease soils affected by soil treatments.**

| **Phyla** | **Class** | **Family** | **Genus** | **Kle** | | | **Alv** | | |
| --- | --- | --- | --- | --- | --- | --- | --- | --- | --- |
|  |  |  |  | **Con** | **H50** | **Gamma** | **Con** | **H50** | **Gamma** |
| ***Acidobacteria*** |  |  |  | **14.7±0a** | **10.2±1b** | **5.9±0c** | **12.4±2a** | **9.3±1b** | **9.8±1b** |
|  | *Acidobacteria_Gp1* | *Edaphobacter* | *Edaphobacter* | 0±0 | 0.1±0 | 0.1±0 | 0.1±0a | 0.3±0b | 0.1±0ab |
|  | *Acidobacteria_Gp1* | *Gp1* | *Gp1* | 3.9±0a | 3.5±0a | 1.7±0b | 3.4±1 | 3.3±1 | 2.9±1 |
|  | *Acidobacteria_Gp1* | *Granulicella* | *Granulicella* | 0±0 | 0±0 | 0±0 | 0.1±0a | 0±0a | 0.4±1b |
|  | *Acidobacteria_Gp13* | *Gp13* | *Gp13* | 0.1±0a | 0±0b | 0±0b | 0.1±0 | 0±0 | 0±0 |
|  | *Acidobacteria_Gp14* | *Gp14* | *Gp14* | 0±0a | 0.2±0b | 0.1±0ab | 0.5±0 | 0.5±0 | 0.4±0 |
|  | *Acidobacteria_Gp16* | *Gp16* | *Gp16* | 1.6±0a | 1.4±0a | 0.5±0b | 0.8±0a | 0.6±0a | 0.3±0b |
|  | *Acidobacteria_Gp2* | *Gp2* | *Gp2* | 1.8±0a | 0.9±0b | 0.4±0c | 1.8±1a | 0.6±0b | 0.7±0b |
|  | *Acidobacteria_Gp3* | *Gp3* | *Gp3* | 3.7±0a | 2.2±0b | 1.2±0c | 3±1a | 2.1±1b | 1.3±0c |
|  | *Acidobacteria_Gp4* | *Gp4* | *Gp4* | 0.8±0a | 0.6±0ab | 0.4±0b | 0.1±0 | 0.2±0 | 0±0 |
|  | *Acidobacteria_Gp5* | *Gp5* | *Gp5* | 0.3±0a | 0±0b | 0.1±0b | 0.1±0 | 0.1±0 | 0±0 |
|  | *Acidobacteria_Gp6* | *Gp6* | *Gp6* | 1.1±0a | 0.4±0b | 0.5±0b | 0.3±0 | 0.2±0 | 0.2±0 |
|  | *Acidobacteria_Gp7* | *Gp7* | *Gp7* | 0.5±0a | 0.3±0b | 0.1±0b | 0.2±0 | 0.2±0 | 0.2±0 |
|  | *Holophagae* | *Holophagaceae* | | 0±0 | 0±0 | 0.1±0 | 0.4±0a | 0±0b | 0.1±0b |
| ***Actinobacteria*** |  |  |  | **13±1a** | **13.8±1a** | **7.9±0b** | **13.9±1a** | **13.2±1a** | **9.8±2b** |
|  | *Actinobacteria* | *Acidimicrobiaceae* | *Ilumatobacter* | 0.4±0a | 0.1±0b | 0±0b | 0.1±0 | 0±0 | 0±0 |
|  | *Actinobacteria* | *Geodermatophilaceae* | *Blastococcus* | 0.2±0a | 0.5±0b | 0.1±0a | 0.1±0ab | 0.2±0a | 0±0b |
|  | *Actinobacteria* | *Intrasporangiaceae* | | 0.4±0 | 0.4±0 | 0.2±0 | 0.8±0a | 0.7±0a | 0.2±0b |
|  | *Actinobacteria* | *Microbacteriaceae* | | 0.1±0a | 0.2±0ab | 0.2±0b | 0.2±0a | 0.2±0a | 1.2±1b |
|  | *Actinobacteria* | *Micrococcaceae* | *Arthrobacter* | 1.1±0 | 0.8±0 | 0.9±0 | 0.9±1a | 1±1a | 1.8±0b |
|  | *Actinobacteria* | *Micromonosporaceae* | *Dactylosporangium* | 0±0 | 0±0 | 0±0 | 0±0 | 0.1±0 | 0±0 |
|  | *Actinobacteria* | *Mycobacteriaceae* | *Mycobacterium* | 0±0 | 0.1±0 | 0±0 | 0.3±0a | 0.2±0ab | 0.1±0b |
|  | *Actinobacteria* | *Nakamurellaceae* | | 0.1±0 | 0±0 | 0±0 | 0.2±0a | 0±0ab | 0±0b |
|  | *Actinobacteria* | *Nocardioidaceae* | | 1.4±0 | 1.1±0 | 1.2±0 | 0.9±0a | 0.7±0a | 0.3±0b |
|  | *Actinobacteria* | *Nocardioidaceae* | *Nocardioides* | 0.9±0 | 0.8±0 | 0.8±0 | 0.7±0a | 0.6±0a | 0.2±0b |
|  | *Actinobacteria* | *Pseudonocardiaceae* | *Pseudonocardia* | 0.2±0 | 0.1±0 | 0.1±0 | 0.3±0a | 0.1±0b | 0.1±0b |
|  | *Actinobacteria* | *Streptomycetaceae* | *Streptomyces* | 0.1±0a | 0.6±0b | 0.2±0a | 0.2±0a | 0.5±0b | 0.2±0a |
|  | *Actinobacteria* |  |  | 1.2±0a | 1.4±0a | 0.3±0b | 1.3±0a | 1.6±0a | 0.2±0b |
| ***Bacteroidetes*** |  |  |  | **3.8±0a** | **4.2±1a** | **5.5±1b** | **3.7±2a** | **1.9±1b** | **4.8±1c** |
|  | *Bacteroidetes_*  *incertae_sedis* | *Ohtaekwangia* | *Ohtaekwangia* | 0.1±0ab | 0±0a | 0.2±0b | 0.1±0 | 0±0 | 0±0 |
|  | *Sphingobacteria* | *Chitinophagaceae* | | 2.4±0a | 3.4±1b | 3.5±1b | 2.6±1a | 1.2±0b | 3.2±1c |
|  | *Sphingobacteria* | *Chitinophagaceae* | *Niastella* | 0±0a | 0.3±0b | 0±0a | 0±0 | 0±0 | 0±0 |
|  | *Sphingobacteria* | *Cytophagaceae* | | 0.1±0ab | 0±0a | 0.2±0b | 0±0 | 0±0 | 0±0 |
|  | *Sphingobacteria* | *Sphingobacteriaceae* | | 0.2±0a | 0.2±0a | 0.6±0b | 0.2±0a | 0.1±0a | 0.7±0b |
|  | *Sphingobacteria* | *Sphingobacteriaceae* | *Mucilaginibacter* | 0.1±0a | 0.1±0a | 0.3±0b | 0.1±0a | 0±0a | 0.5±0b |
|  | *Sphingobacteria* | *Sphingobacteriaceae* | *Pedobacter* | 0.1±0 | 0.1±0 | 0.2±0 | 0±0 | 0±0 | 0±0 |
| ***Chloroflexi*** |  |  |  | **0.4±0a** | **0.2±0b** | **0.3±0ab** | **0.4±0** | **0.4±0** | **0.2±0** |
|  | *Anaerolineae* | *Anaerolineaceae* | | 0.3±0a | 0.1±0b | 0.2±0ab | 0.2±0a | 0.1±0ab | 0±0b |
| ***Cyanobacteria/***  ***Chloroplast*** |  |  |  | **1.6±1a** | **0.5±0b** | **0.5±0b** | **2.4±1a** | **3.3±2b** | **1.7±1c** |
|  | *Chloroplast* | *Chloroplast* | *Bacillariophyta* | 1±1a | 0.4±0b | 0.4±0b | 1.3±1a | 1.9±2b | 1.1±1a |
|  | *Chloroplast* | *Chloroplast* | *Streptophyta* | 0.3±0a | 0±0b | 0.1±0b | 0.8±1a | 1.3±1b | 0.5±0a |
|  | *Cyanobacteria* |  |  | 0.1±0a | 0±0b | 0±0b | 0.1±0 | 0±0 | 0±0 |
| ***Deinococcus-Thermus*** | *Deinococci* | *Deinococcaceae* | *Deinococcus* | **0±0a** | **0±0a** | **0.3±0b** | **0±0** | **0±0** | **0.1±0** |
| ***Firmicutes*** |  |  |  | **21.1±3a** | **26.6±1b** | **28.2±1c** | **28±11a** | **34.5±7b** | **22.9±7c** |
|  | *Bacilli* | *Bacillaceae 1* | *Bacillus* | 4.1±1a | 5.1±0b | 5.5±0b | 11.1±4a | 13.2±3b | 7.6±2c |
|  | *Bacilli* | *Bacillaceae 2* | *Tuberibacillus* | 0.2±0a | 0±0b | 0±0b | 0±0 | 0±0 | 0±0 |
|  | *Bacilli* | *Paenibacillaceae 1* | *Brevibacillus* | 0.1±0a | 0.4±0b | 0.1±0a | 0.1±0 | 0.1±0 | 0.1±0 |
|  | *Bacilli* | *Paenibacillaceae 1* | *Paenibacillus* | 1.4±0a | 2.1±0b | 1.6±0a | 1.4±0a | 2.5±1b | 1.7±1a |
|  | *Bacilli* | *Paenibacillaceae 2* | *Aneurinibacillus* | 0±0a | 0.3±0b | 0±0a | 0±0 | 0±0 | 0±0 |
|  | *Bacilli* | *Planococcaceae* | *Ureibacillus* | 0.1±0 | 0.1±0 | 0.2±0 | 0±0 | 0±0 | 0±0 |
|  | *Bacilli* | *Thermoactinomycetaceae 1* | *Thermoactinomyces* | 0.4±0 | 0.3±0 | 0.4±0 | 0.1±0 | 0.2±0 | 0.1±0 |
|  | *Bacilli* | *Thermoactinomycetaceae 2* | *Planifilum* | 0.5±0 | 0.4±0 | 0.6±0 | 0±0 | 0±0 | 0±0 |
|  | *Clostridia* | *Clostridiaceae 1* | *Clostridium sensu stricto* | 0.9±0 | 0.9±0 | 1±0 | 2±1a | 2.4±1a | 0.7±0b |
|  | *Clostridia* | *Clostridiales_Incertae Sedis XVIII* | *Symbiobacterium* | 0.1±0 | 0.1±0 | 0.2±0 | 0±0 | 0±0 | 0±0 |
|  | *Clostridia* | *Lachnospiraceae* | | 0.5±0 | 0.5±0 | 0.7±0 | 0.3±0 | 0.3±0 | 0.2±0 |
|  | *Clostridia* | *Peptostreptococcaceae* | | 0.6±0a | 0.5±0a | 1±0b | 0.5±0 | 0.6±0 | 0.5±0 |
|  | *Clostridia* | *Peptostreptococcaceae* | *Clostridium XI* | 0.6±0a | 0.5±0a | 1±0b | 0.5±0 | 0.6±0 | 0.5±0 |
|  | *Clostridia* | *Ruminococcaceae* | *Clostridium III* | 0.3±0 | 0.3±0 | 0.5±0 | 0.3±0a | 0.3±0a | 0.1±0b |
|  | *Erysipelotrichia* | *Erysipelotrichaceae* | *Turicibacter* | 0.4±0 | 0.3±0 | 0.4±0 | 0.2±0 | 0.3±0 | 0.3±0 |
|  | *Negativicutes* | *Veillonellaceae* | *Psychrosinus* | 0.1±0 | 0.1±0 | 0.1±0 | 0.3±0 | 0.3±0 | 0.2±0 |
| ***Gemmatimonadetes*** | *Gemmatimonadetes* | *Gemmatimonadaceae* | *Gemmatimonas* | **2.6±0a** | **4.1±1b** | **5.2±1c** | **1.7±0** | **1.8±1** | **1.7±1** |
| ***Nitrospira*** | *Nitrospira* | *Nitrospiraceae* | *Nitrospira* | **0.7±0a** | **0±0b** | **0±0b** | **0.6±0a** | **0.1±0b** | **0±0b** |
| ***Proteobacteria*** |  | |  | **30.5±2a** | **28.6±0b** | **36.5±1c** | **28.8±7a** | **26.4±2a** | **42.6±8b** |
|  | *Alphaproteobacteria* | |  | 18.3±2a | 17.7±1a | 21.3±1b | 14.6±5a | 14.6±2a | 20.4±1b |
|  | *Alphaproteobacteria* | *Caulobacteraceae* | | 0.6±0a | 1.5±1b | 1.2±0b | 0.5±0a | 1±0b | 1.3±1b |
|  | *Alphaproteobacteria* | *Caulobacteraceae* | *Phenylobacterium* | 0.4±0a | 1.1±0b | 0.6±0c | 0.3±0a | 0.6±0b | 0.6±0b |
|  | *Alphaproteobacteria* | *Bradyrhizobiaceae* | | 1.6±0a | 1±0b | 1.9±0a | 1.7±1a | 1±0b | 2.1±1c |
|  | *Alphaproteobacteria* | *Hyphomicrobiaceae* | | 1±0ab | 0.8±0a | 1.3±0b | 0.7±0a | 0.5±0a | 1.1±0b |
|  | *Alphaproteobacteria* | *Hyphomicrobiaceae* | *Devosia* | 0.3±0a | 0.2±0a | 0.8±0b | 0.3±0a | 0.1±0a | 0.8±0b |
|  | *Alphaproteobacteria* | *Methylobacteriaceae* | | 0.1±0a | 0.2±0b | 1.3±0c | 0.1±0 | 0.1±0 | 0±0 |
|  | *Alphaproteobacteria* | *Methylobacteriaceae* | *Microvirga* | 0±0a | 0.2±0b | 1.3±0c | 0±0 | 0.1±0 | 0±0 |
|  | *Alphaproteobacteria* | *Methylocystaceae* | | 0.1±0 | 0±0 | 0.1±0 | 0.2±0 | 0.1±0 | 0.1±0 |
|  | *Alphaproteobacteria* | *Phyllobacteriaceae* | | 0.3±0a | 0.1±0b | 0.5±0c | 0.1±0ab | 0±0a | 0.3±0b |
|  | *Alphaproteobacteria* | *Phyllobacteriaceae* | *Mesorhizobium* | 0.3±0a | 0.1±0b | 0.5±0a | 0.1±0ab | 0±0a | 0.3±0b |
|  | *Alphaproteobacteria* | *Acetobacteraceae* | | 0.4±0 | 0.3±0 | 0.4±0 | 1.4±0a | 0.6±0b | 2.4±2c |
|  | *Alphaproteobacteria* | *Acetobacteraceae* | *Acidocella* | 0±0 | 0±0 | 0±0 | 0.6±0a | 0.1±0b | 0.9±1a |
|  | *Alphaproteobacteria* | *Erythrobacteraceae* | | 0±0a | 0.1±0ab | 0.2±0b | 0±0 | 0±0 | 0±0 |
|  | *Alphaproteobacteria* | *Sphingomonadaceae* | | 3.1±0a | 4.3±0b | 3.7±1b | 1.7±2a | 3.6±1b | 3.1±0b |
|  | *Alphaproteobacteria* | *Sphingomonadaceae* | *Novosphingobium* | 0.4±0 | 0.4±0 | 0.5±0 | 0.3±1 | 0.2±0 | 0.2±0 |
|  | *Alphaproteobacteria* | *Sphingomonadaceae* | *Sphingomonas* | 1.7±0a | 2.7±0b | 1.8±0a | 0.7±1a | 2.6±1b | 1.7±0c |
|  | *Betaproteobacteria* | |  | 4.5±0a | 5.1±0b | 6.7±1c | 4.5±1a | 4.1±1a | 6.6±1b |
|  | *Betaproteobacteria* | *Alcaligenaceae* |  | 0.1±0a | 0.5±0b | 0.5±0b | 0.6±0a | 0.9±1ab | 1.3±1b |
|  | *Betaproteobacteria* | *Burkholderiaceae* | *Burkholderia* | 0.1±0a | 0.6±0b | 1.3±0c | 0.5±0 | 0.8±0 | 0.8±0 |
|  | *Betaproteobacteria* | *Comamonadaceae* | | 0.4±0a | 0.7±0a | 1.8±0b | 1±0a | 0.6±0b | 1.9±1c |
|  | *Betaproteobacteria* | *Comamonadaceae* | *Ramlibacter* | 0±0a | 0.2±0b | 0.4±0c | 0±0 | 0±0 | 0.1±0 |
|  | *Betaproteobacteria* | *Oxalobacteraceae* | | 0.3±0a | 0.8±0b | 0.9±0b | 0.2±0a | 0.6±0b | 0.6±0b |
|  | *Betaproteobacteria* | *Nitrosomonadaceae* | | 0.2±0ab | 0.1±0a | 0.3±0b | 0.2±0a | 0±0b | 0.3±0a |
|  | *Betaproteobacteria* | *Nitrosomonadaceae* | *Nitrosospira* | 0.2±0ab | 0.1±0a | 0.3±0b | 0.2±0ab | 0±0a | 0.3±0b |
|  | *Deltaproteobacteria* | |  | 2.5±0 | 2.1±0 | 2.2±0 | 2±0a | 1.8±1ab | 1.4±0b |
|  | *Deltaproteobacteria* | *Geobacteraceae* | *Geobacter* | 0.2±0a | 0±0b | 0.2±0ab | 0.1±0 | 0.1±0 | 0±0 |
|  | *Deltaproteobacteria* | *Polyangiaceae* |  | 0.2±0 | 0.2±0 | 0.2±0 | 0.1±0 | 0.1±0 | 0.1±0 |
|  | *Gammaproteobacteria* | |  | 4.2±0a | 3±1b | 5.4±0c | 6.8±1a | 5.5±2b | 13.6±7c |
|  | *Gammaproteobacteria* | *Coxiellaceae* | *Aquicella* | 0.2±0 | 0.2±0 | 0.1±0 | 0.1±0 | 0.1±0 | 0±0 |
|  | *Gammaproteobacteria* | *Methylococcaceae* | | 0.3±0 | 0.2±0 | 0.3±0 | 0.2±0a | 0.1±0b | 0±0b |
|  | *Gammaproteobacteria* | *Methylococcaceae* | *Methylobacter* | 0.2±0 | 0.1±0 | 0.1±0 | 0.2±0a | 0.1±0b | 0±0b |
|  | *Gammaproteobacteria* | *Xanthomonadaceae* | | 1.8±0a | 1.6±1a | 3.7±0b | 4.6±0a | 4.1±2a | 11.7±7b |
|  | *Gammaproteobacteria* | *Xanthomonadaceae* | *Arenimonas* | 0.1±0a | 0±0a | 0.6±0b | 0±0 | 0±0 | 0±0 |
|  | *Gammaproteobacteria* | *Xanthomonadaceae* | *Dokdonella* | 0.5±0a | 0.2±0b | 0.1±0b | 0.4±0a | 0.1±0b | 0.1±0b |
|  | *Gammaproteobacteria* | *Xanthomonadaceae* | *Dyella* | 0.2±0a | 0±0b | 0.2±0a | 0.2±0 | 0.2±0 | 0±0 |
|  | *Gammaproteobacteria* | *Xanthomonadaceae* | *Lysobacter* | 0.2±0a | 0.3±0ab | 0.4±0b | 0.1±0ab | 0±0a | 0.2±0b |
|  | *Gammaproteobacteria* | *Xanthomonadaceae* | *Rhodanobacter* | 0.2±0a | 0.2±0a | 1±0b | 0.8±1a | 0.8±0a | 3.6±1b |
| ***TM7*** | *TM7_genera_incertae_*  *sedis* | *TM7_genera_incertae_*  *sedis* | *TM7_genera_*  *incertae_sedis* | **0.2±0a** | **0.2±0a** | **0.5±0b** | **0.2±0ab** | **0.1±0a** | **0.4±0b** |
| ***Verrucomicrobia*** |  |  |  | **0.3±0a** | **0.1±0b** | **0.2±0ab** | **0.3±0** | **0.2±0** | **0.2±0** |
| ***WS3*** | *WS3_genera_incertae_*  *sedis* | *WS3_genera_incertae_*  *sedis* | *WS3_genera_*  *incertae_sedis* | **0.2±0a** | **0±0b** | **0±0b** | **0.2±0a** | **0±0b** | **0±0b** |

(Average relative abundances±SD), significant differences in relative abundances between treatments within soil type at genus level were indicated by different letters, Tukey test, p < 0.05 and n = 4. Significant increases in relative abundances compared to Con are highlighted in green, while significant decreases are highlighted in orange.

**S4 Table. Number of analyzed sequences, observed diversity richness (OTUs), ‘invsimpson' diversity index and the sample coverage of the 16S rRNA genes detected in soil TC-DNA of different RD soil treatments.**

| **Soil** | **Treatment** | **Reads** | **OTU** | **Invsimpson** | **Coverage** |
| --- | --- | --- | --- | --- | --- |
| **Kle** | **KleCon** | 8092±1706 | 1285±143 a | 44.0±7.0 a | 0.92±0.01 a |
|  | **KleH50** | 8539±1008 | 1037±80 a | 34.9±1.3 a | 0.95±0.01 b |
|  | **KleGamma** | 8594±1637 | 1116±105 a | 36.0±1.9 a | 0.94±0.01 ab |
| **Alv** | **AlvCon** | 7020±2374 | 751±149 b | 16.3±11.6 b | 0.95±0.01 b |
|  | **AlvH50** | 7102±2016 | 655±90 b | 11.4±2.0 b | 0.96±0.01 b |
|  | **AlvGamma** | 7555±1489 | 632±104 b | 14.2±0.9 b | 0.96±0.0 b |

(Average±SD), different letters indicate significant differences between the soil treatments, Tukey test, p < 0.05 and n = 4.

**
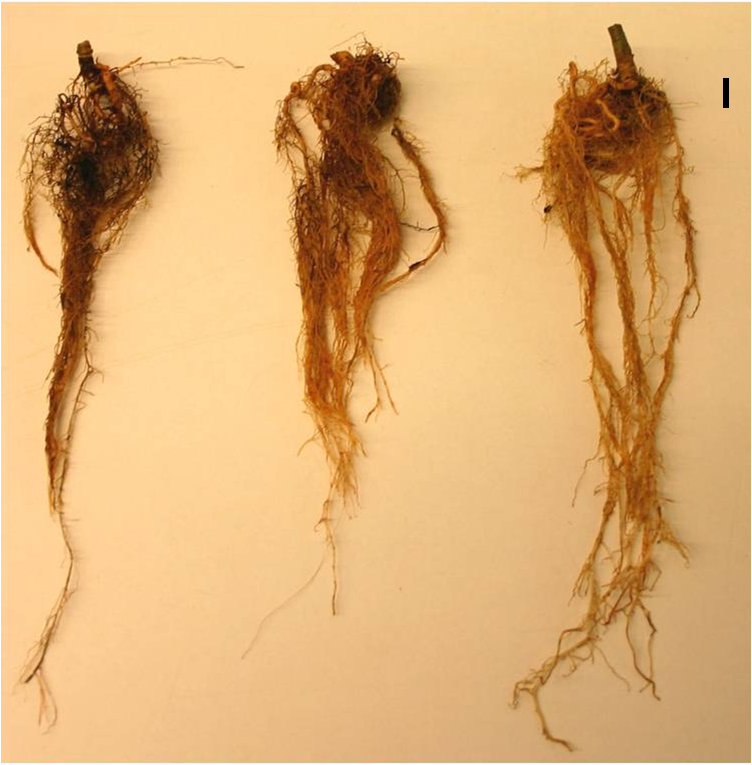
**

A

**
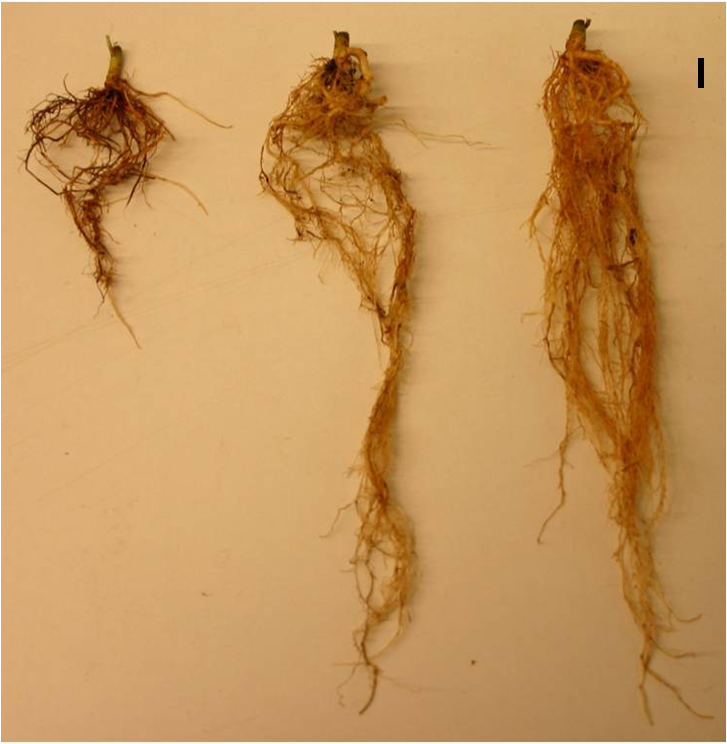
**

B

**S1 Figure. Roots of M26 plants after growing for eight weeks in different replant disease soil treatments, in Kle (A) and Alv (B) soils.** Con (left), H50 (middle) and Gamma (right). Bar indicates 10 cm.

**Reference**

Van Reeuwijk, L.P. (2002). Procedures for soil analysis (6th edition). *Technical Paper, ISRIC, Wageningen* 9. Available: http://www.isric.org/content/technical-papers16-1.
